# World Health Organization simulations: an increasingly popular learning tool for the development of future global health practitioners

**DOI:** 10.7189/jogh.10.010305

**Published:** 2020-06

**Authors:** Brian LH Wong, Mark P Khurana, Neha Acharya, Michalina Drejza, Diogo Martins

**Affiliations:** 1 Medical Research Council Unit for Lifelong Health and Ageing at UCL, Department of Population Science and Experimental Medicine, UCL Institute of Cardiovascular Science, University College London, UK; 2 Rigshospitalet, University of Copenhagen, CHIP, Department of Infectious Diseases, Section 2100, Copenhagen, Denmark; 3 Abt Associates, Rockville, MD, USA; 4 London School of Hygiene and Tropical Medicine, London, UK; * Equal authorship.

Interest among students and young professionals (SYPs) in global health is undoubtedly on the rise [1,2]. Novel methods for increasing engagement of SYPs with the field are imperative. An increasingly interconnected and globalized world poses an array of new health challenges, including a rising burden of NCDs, the rapid movement of communicable diseases and threats to global health security. Such pressing issues demand that future health professionals develop a more holistic and interdisciplinary understanding of health and the necessary critical skills of policy-making, negotiation, and conflict-resolution. To quote Professor Ilona Kickbusch of the Graduate Institute of International and Development Studies in Geneva, “global health diplomacy is gaining in importance and its negotiators should be well prepared” [3]. One such initiative with this ethos and which provides this cadre of proficiencies is student-led Model World Health Organization (WHO) simulations.

## SIMULATIONS AS A LEARNING TOOL

Model WHO simulations recreate the proceedings of the World Health Assembly (WHA), the decision-making body of the WHO, which takes place annually in Geneva, Switzerland. In these simulations, participants discuss topics within a pre-defined theme for the conference (**Table 1**); previous themes include *Sexual Health*; *Women’s, Children’s and Adolescents’ Health*; and *Environmental Health*. Simulation participants are designated roles from a diverse list of stakeholders: Member States, Non-State Actors (NSAs) – such as non-governmental organizations or private sector representatives, UN Agencies and Media/Press. Member State representatives discuss and negotiate policy papers, initially within their given WHO region [4], and later collectively in plenary. During plenary sessions, all participants assemble to merge and amend draft resolutions, with the ultimate goal of passing a final conference resolution. Ideally, the final resolution reflects how Member States prioritize and develop solutions to address the overall conference theme – similar to WHO resolutions at the WHA. Throughout the simulation process, NSA representatives seek to influence discussions, offer recommendations, and capture discourse through written/video formats.

**Table 1:** List of locations with student-run Model World Health Organization Simulations in 2018, themes and attendance

Region	Location	Theme	Attendance
Europe	London	Improving the Health of Women, Children and Adolescents	175
	Paris	Environmental Health	120
	Copenhagen	Access to essential medicines - How can we ensure global equality?	80
	Sheffield	Outbreaks and Pandemics: Addressing the Next Crisis	87
North America	Montreal	Health Crises	80
	Toronto	Sexual Health	50
	Edmonton	Organ Trade & Trafficking	35
	Newfoundland and Labrador	Critical and Acute Care	63
	Chapel Hill, North Carolina	Global Health Innovation: Novel Idea, New Horizons	112
	Blacksburg, Virginia	Vaccines	65
	Norman, Oklahoma	Impacting Health Through Education	83
	Baltimore, Maryland	Social Sustainability	71
Australia	Melbourne	Mental Health: Beyond the Stigma	65
Asia	Tokyo	Creating International Health Regulations for Emerging and Re-emerging Infectious Diseases	50

**Figure:**
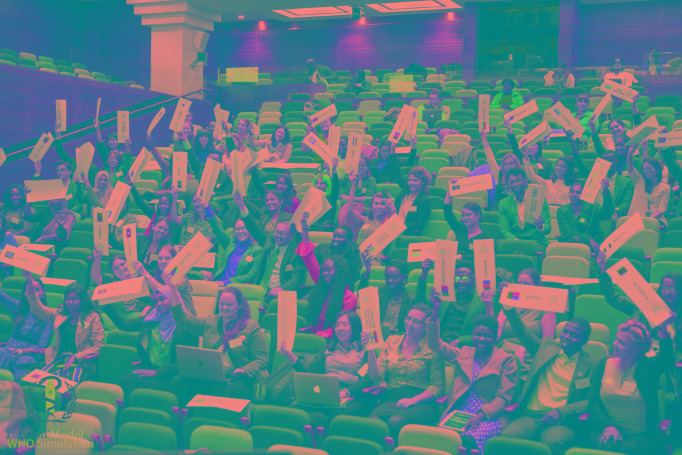
Photo: from the 2019 LonWHO conference, held at Bush House, King’s College, London (from the collection of Charlie Harless, used with permission).

## ADAPTING TO DIFFERENT FORMATS

Alternative versions of these simulations have in fact been adopted in other settings. At University College London (UCL), a five-day model WHO simulation has been integrated into the “Outbreak! Infectious Diseases” strand of the annual UCL Global Citizenship Program [5]. In addition, at the biennial Young Leaders for Health (YLH) conference in Berlin, a WHO simulation has been included in the official conference program since 2015 [6]. To cater to the growing interest in these types of simulations, regional organizations have been established to standardize simulation formats and promote knowledge-sharing between simulation organizers; these include the American Mock World Health Organization (AMWHO) and the United Kingdom Model World Health Organization (UKWHO) [7,8].

## CREATIVE MODULES IN GLOBAL HEALTH

These conferences provide SYPs with experiential learning environments to engage with global health issues, undertake research, and partake in negotiations surrounding global health policy. At the most recent WHO simulation in London, Lon WHO (n = 87 responses), 87.4% of participants considered a career in global public health prior to the conference compared to 96.6% after the conference. In a similar vein, 98.9% of participants would recommend the conference to peers. Model WHO simulations also have the capacity to complement other creative learning modules in global health such as MOOCs, webinars, and social media initiatives [9]. Furthermore, they allow for SYPs from all disciplines – spanning from nursing to health economics to political science – to engage in stimulating debate, fostering a truly interdisciplinary approach to health in true WHO spirit. In doing so, simulations allow for innovative and collaborative thinking to surface, whilst providing SYPs with a more practical and real-world experience in global health diplomacy to complement standardized taught curricula. Promotion and expansion of creative learning modules such as WHO simulations, both in terms of volume and geographic reach, has the potential to inspire and engage future global health practitioners in developing the necessary technical knowledge and soft skills essential for global health diplomacy.
